# Smartphone App–Delivered Mindfulness-Based Intervention for Mild Traumatic Brain Injury in Adolescents: Protocol for a Feasibility Randomized Controlled Trial

**DOI:** 10.2196/57226

**Published:** 2024-04-11

**Authors:** Andrée-Anne Ledoux, Roger Zemek, Molly Cairncross, Noah Silverberg, Veronik Sicard, Nicholas Barrowman, Gary Goldfield, Clare Gray, Ashley D Harris, Natalia Jaworska, Nick Reed, Bechara J Saab, Andra Smith, Lisa Walker

**Affiliations:** 1 Children's Hospital of Eastern Ontario Research Institute Ottawa, ON Canada; 2 Department of Cellular Molecular Medicine Faculty of Medicine University of Ottawa Ottawa, ON Canada; 3 School of Psychology Faculty of Social Sciences University of Ottawa Ottawa, ON Canada; 4 University of Ottawa Brain and Mind Research Institute University of Ottawa Ottawa, ON Canada; 5 Department of Pediatrics Faculty of Medicine University of Ottawa Ottawa, ON Canada; 6 Department of Psychology Simon Fraser University Burnaby, BC Canada; 7 BC Children’s Hospital Research Institute Vancouver, BC Canada; 8 Department of Psychology University of British Columbia Vancouver, BC Canada; 9 Rehabilitation Research Program Centre for Aging SMART Vancouver Coastal Health Research Institute Vancouver, BC Canada; 10 Children's Hospital of Eastern Ontario Ottawa, ON Canada; 11 Department of Psychiatry Faculty of Medicine University of Ottawa Ottawa, ON Canada; 12 Department of Radiology Hotchkiss Brain Institute Alberta Children’s Hospital Research Institute, University of Calgary Calgary, AB Canada; 13 Royal Ottawa Mental Health Centre Ottawa, ON Canada; 14 Department of Occupational Science & Occupational Therapy University of Toronto Toronto, ON Canada; 15 Mobio Interactive Singapore Singapore; 16 The Ottawa Hospital Ottawa, ON Canada

**Keywords:** pediatric, concussion, persisting symptoms after concussion, mindfulness, randomized controlled trial, feasibility RCT, psychological intervention, youth, digital therapeutics, eHealth, mobile health, mHealth, mobile phone

## Abstract

**Background:**

Concussion in children and adolescents is a significant public health concern, with 30% to 35% of patients at risk for prolonged emotional, cognitive, sleep, or physical symptoms. These symptoms negatively impact a child’s quality of life while interfering with their participation in important neurodevelopmental activities such as schoolwork, socializing, and sports. Early psychological intervention following a concussion may improve the ability to regulate emotions and adapt to postinjury symptoms, resulting in the greater acceptance of change; reduced stress; and recovery of somatic, emotional, and cognitive symptoms.

**Objective:**

The primary objective of this study is to assess the feasibility of conducting a parallel-group (1:1) randomized controlled trial (RCT) to evaluate a digital therapeutics (DTx) mindfulness-based intervention (MBI) in adolescents aged 12 to <18 years. The attention-matched comparator intervention (a math game also used in previous RCTs) will be delivered on the same DTx platform. Both groups will be provided with the standard of care guidelines. The secondary objective is to examine intervention trends for quality of life; resilience; self-efficacy; cognition such as attention, working memory, and executive functioning; symptom burden; and anxiety and depression scores at 4 weeks after concussion, which will inform a more definitive RCT. A subsample will be used to examine whether those randomized to the experimental intervention group have different brain-based imaging patterns compared with those randomized to the control group.

**Methods:**

This study is a double-blind Health Canada–regulated trial. A total of 70 participants will be enrolled within 7 days of concussion and randomly assigned to receive the 4-week DTx MBI (experimental group) or comparator intervention. Feasibility will be assessed based on the recruitment rate, treatment adherence to both interventions, and retention. All outcome measures will be evaluated before the intervention (within 7 days after injury) and at 1, 2, and 4 weeks after the injury. A subset of 60 participants will undergo magnetic resonance imaging within 72 hours and at 4 weeks after recruitment to identify the neurophysiological mechanisms underlying the potential benefits from MBI training in adolescents following a concussion.

**Results:**

The recruitment began in October 2022, and the data collection is expected to be completed by September 2024. Data collection and management is still in progress; therefore, data analysis is yet to be conducted.

**Conclusions:**

This trial will confirm the feasibility and resolve uncertainties to inform a future definitive multicenter efficacy RCT. If proven effective, a smartphone-based MBI has the potential to be an accessible and low-risk preventive treatment for youth at risk of experiencing prolonged postconcussion symptoms and complications.

**Trial Registration:**

ClinicalTrials.gov NCT05105802; https://classic.clinicaltrials.gov/ct2/show/NCT05105802

**International Registered Report Identifier (IRRID):**

DERR1-10.2196/57226

## Introduction

### Background and Rationale

Approximately 30% to 35% of the children and adolescents who experience a concussion are considered to have persisting symptoms after concussion (PSAC) [1,2], defined as the persistence of somatic (eg, headache, dizziness, and fatigue); cognitive (eg, memory, concentration, and confusion); and other physical, psychological, and behavioral changes lasting beyond 1 month [3]. PSAC may impair daily activities including schoolwork, socializing, and sports and thereby reduce the quality of life (QoL) [4]. Studies have demonstrated that both noninjury and injury factors predict PSAC following a concussion [2,5,6], with the contribution of injury factors to PSAC decreasing over time. However, premorbid conditions such as preinjury somatic symptoms [7], migraine [2], preinjury cognitive ability [8], preexisting attention and mood concerns [9], anxiety [10], and coping strategies [11] remain important predictors of symptom persistence over time [8,12]. Preventive, early rehabilitation programs such as interventions focusing on building psychological resiliency, emotional regulation, and self-efficacy might be key to managing concussions, reducing PSAC risk, and promoting neural recovery.

Mindfulness-based interventions (MBIs) are *present-centered* interventions that encourage acceptance of thoughts and emotions as they occur in the moment without judgment. MBIs have been demonstrated to improve attentional focus, cognitive flexibility, depression and anxiety symptoms, self-concept, resiliency, and academic achievements as well as reduce affective reactivity and fear in clinical youth samples [13]. In addition, in-person MBIs seem effective at treating preinjury predictors of PSAC, such as mood disorders [14-17], somatization-related illness [14,18,19], headache disorders [20], and chronic pain [21]. However, in-person interventions may not be suitable for adolescents with acute concussion owing to high cost and low accessibility [22,23] and require commitment from parents and children for in-person weekly meetings for periods varying from 8 to 16 weeks. Brief (as short as 3 days to 4 weeks) web-based MBIs with guided meditation have been shown to have positive effects on anxiety, negative mood, perceived stress, and attention in different clinical populations [24-27]. MBIs may foster adaptive coping, increase resiliency, and reduce the risk of PSAC following pediatric concussion. Smartphone, or mobile, apps may present a versatile and personalized platform for the delivery of a dedicated MBI for populations experiencing a concussion [28].

Health care digital therapeutics (DTx; smartphone app) have gained popularity in the past decade as a means of screening patients, monitoring symptoms, and delivering psychoeducation and interventions [28,29]. The efficacy of mobile apps in delivering psychological interventions has been demonstrated in mental health samples, particularly in the treatment of depression and anxiety. Randomized controlled trials (RCTs) have shown that app-based interventions can effectively improve depressive and anxiety symptoms compared with waiting list, alternative care, or control conditions [29-34]. Similarly, a recent meta-analysis of RCTs on app-based MBIs has demonstrated significant small-to-medium effects in improving perceived stress, symptoms of depression and anxiety, QoL, psychological well-being, and positive affects, compared with control conditions, waitlist, and alternative interventions [35]. However, the application of an app-based MBI in pediatric concussion has not yet been studied.

We partnered with Mobio Interactive Inc (Toronto, Ontario, Canada), the creator of a clinically validated platform called *AmDTx*, to create an app-based dedicated DTx MBI for pediatric concussion. We assessed the acceptability and usability of the DTx with a small open-label single-arm study (N=10) [36]. The participants reported high satisfaction and found the app easy to use and the treatment to be credible. They also developed a strong alliance with their MBI guides (ie, those who are leading the meditation and psychoeducation sessions) [37]. The primary aim of this study is to assess the feasibility of the 4-week planned RCT methods to determine whether we can advance to an efficacy trial and, thereby, elevate our understanding of the optimal time points for collecting outcome measures. At 4 weeks, those randomized to the DTx MBI will have the opportunity to continue for another 4 weeks, that is, completing a total of 8 weeks of MBI. The goal of this additional 4-week intervention is to explore feasibility outcomes in an 8-week intervention. Finally, those who are part of the DTx control group (attention-matched comparator intervention) will have the opportunity to cross over to the DTx MBI group.

### Primary Research Objective

The primary research objectives are to assess the feasibility of conducting a larger RCT by evaluating recruitment efficiency, adherence to treatment, credibility, and retention.

### Secondary Research Objectives

The **s**econdary research objectives were as follows:

1. Establish the participants’ expectancy toward both interventions.
2. Examine the participants’ satisfaction with the interventions.
3. Generate estimates to inform sample size calculations for a future multicenter RCT.
4. Assess whether there is preliminary evidence of an efficacy signal for QoL, fatigue, symptoms, resiliency, self-efficacy, cognition, anxiety, depression, and mindfulness.
5. Examine the experimental group’s baseline characteristics, adherence, and attrition rates in those who chose to continue for an additional 4 weeks compared with those who chose to complete the study at 4 weeks compared with those who were lost to follow-up.
6. Examine the control group’s baseline characteristics, adherence, and attrition rates in those who chose to cross over to the experimental interventions at 4 weeks versus those who chose to complete the study at 4 week versus those who were lost to follow-up.
7. Examine whether the experimental intervention is associated with differential brain-based imaging patterns of functional connectivity, cerebral blood flow, and spectroscopy.
8. Examine safety by examining whether the experimental intervention has increased adverse events (ie, total number) relative to the control intervention.

## Methods

### Trial Design

**Figure 1:**
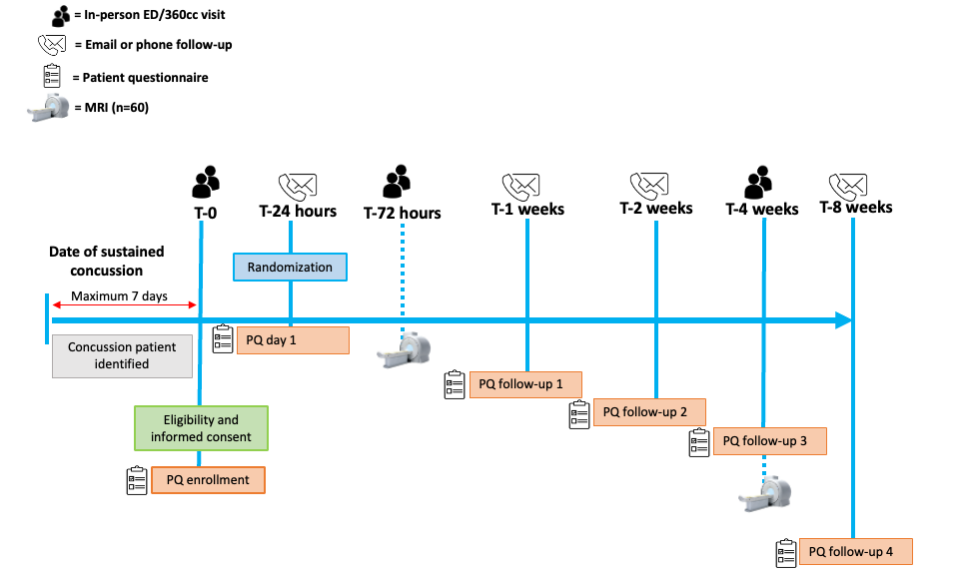
Flowchart of the study procedures. 360cc: 360 Concussion Care; ED: emergency department; MRI: magnetic resonance imaging; PQ: patient questionnaire.

The proposed study is a Health Canada–regulated parallel-group (1:1) RCT, where patients within 7 days of a concussion will be randomly assigned to one of the two groups: (1) experimental group (n=35), early introduction of the DTx MBI program and (2) control group (n=35), sham DTx attention-matched comparator intervention. The participants will start the intervention the next day after being enrolled in the study. After 4 weeks, those in the experimental group will be offered the MBI program with 4-week additional education modules. Those who are part of the control group will be offered to cross over to start the DTx MBI. Of the 70 participants enrolled, 60 (86%) consenting participants (n=30, 50% from the experimental group and n=30, 50% from the control group) will be enrolled in neuroimaging, which requires participation in a brain scanning session within 72 hours of enrollment and during week 4 of the study. For a schematic diagram of the trial design, procedures, and stages, refer to the study flow diagram in Figure 1.

### Study Setting

The study will be conducted at the Children’s Hospital of Eastern Ontario (CHEO) and the 360 Concussion Care (360cc) clinic, a tertiary health care concussion clinic. The neuroimaging component of the study will be completed at the Royal Ottawa Mental Health Centre’s Brain Imaging Centre on a 3-Tesla Siemens magnetic resonance-positron emission tomography system with a 32-channel head coil (Siemens Biograph mMR, Siemens).

### Eligibility Criteria

#### Inclusion Criteria

Adolescents presenting to either CHEO’s emergency department (ED) or 360cc after sustaining a direct or indirect mild traumatic brain injury (TBI) will be eligible to participate in this study if they (1) are aged 12 to <18 years; (2) have a diagnosed concussion (by a physician), (3) presented to the ED or 360cc within 7 days of TBI; (4) have a score >4 on the Predicting and Preventing Post-Concussive Problems in Pediatrics (5P) clinical risk score, a prediction rule to identify those at risk of poorer outcomes at 4 weeks postinjury [2]; and (5) are proficient in English [38]. To increase the probability of including adolescents with a true concussion, the adapted version of the American Congress of Rehabilitation Medicine [39] framework will be used [40]. Those with either 1 *highest level of certainty* symptom (dazed, confused, or trouble thinking within minutes after injury, difficulty remembering what happened just before or minutes after injury, and loss of consciousness) or 2 *higher level of certainty* symptoms (nausea or vomiting, headache, dizziness, clumsy or balance problems, blurred or double or change in vision, difficulty concentrating, and sensitivity to light or noise) immediately or within 1 hour of injury will be included.

#### Exclusion Criteria

Patients will be excluded if they present with TBIs with any of the following: (1) a Glasgow Coma Scale score of ≤13; (2) abnormality on standard neuroimaging studies [41], including positive head computed tomography findings (neuroimaging is not required, but may be performed if clinically indicated); (3) neurosurgical operative intervention, intubation, or intensive care required; (4) multisystem injuries with the treatment requiring hospital admission, operating room, or procedural sedation in ED (hospital admission for observation or management of ongoing concussion symptoms is not an exclusion criteria); (5) severe chronic neurological developmental delay resulting in communication difficulties; (6) intoxication to alcohol or drugs at the time of ED presentation as per clinician judgment; (7) no clear history of trauma as primary event to the concussion (eg, seizure, syncope, and migraine); (8) prior psychiatric hospitalization; (9) inability to obtain written informed consent or assent (eg, language barrier); (10) legal guardian not present (certain forms need to be completed by parents or legal guardians); (11) no internet and mobile or tablet access; and (12) currently in therapy.

In addition, patients will not be included in the magnetic resonance imaging (MRI) component if they present with the following: (1) previous neurological or neurodevelopmental disorder such as epilepsy, intellectual disability, or autism (history of attention-deficit or hyperactivity disorder or learning disability is not exclusionary); (2) previous stroke or transient ischemic attacks; (3) sedation medication (eg, propofol, ketamine, nitrous oxide, midazolam, benzodiazepines, and fentanyl) administered before or during ED visit; (4) inability to be present at the 72-hour (+48 or –48 hours) and 4-week (+5 or –5 days) MRI follow-up visits; and (5) medical contraindications to MRI (eg, claustrophobia, pregnancy, or presence of braces or spacers or other metal implants).

### Randomization, Blinding, and Allocation

#### Sequence Generation

The CHEO Clinical Research Unit will provide data management services for this study and retain randomization codes. The randomization sequence will be created using R software (version 3.1.1; R Foundation for Statistical Computing) [42] and will be stratified by sex, with a 1:1 allocation using random blocks.

#### Allocation Concealment

A statistician at the CHEO CRU (not involved in the study) will maintain the master list.

#### Implementation

The day after enrollment and once all questionnaire data are complete, research assistants (RAs) will call participants over the phone, and RAs will log into REDCap (Research Electronic Data Capture; Vanderbilt University) to obtain the patient’s group allocation. Only the RAs who randomized participants will be unblinded. The research team will be blinded to data acquisition. In case of a serious adverse event, the principal investigator and appropriate research staff will be unblinded to the participant’s group.

### Methods for Protecting Against Bias

The investigators, coaches, other research staff, data management staff, and biostatisticians will be blinded to the group assignment. Patients and families will be blinded to the purpose and expected results of the interventions and to the other treatment arm.

### Intervention Description

#### Experimental Group Description: MBI Training

**Figure 2:**
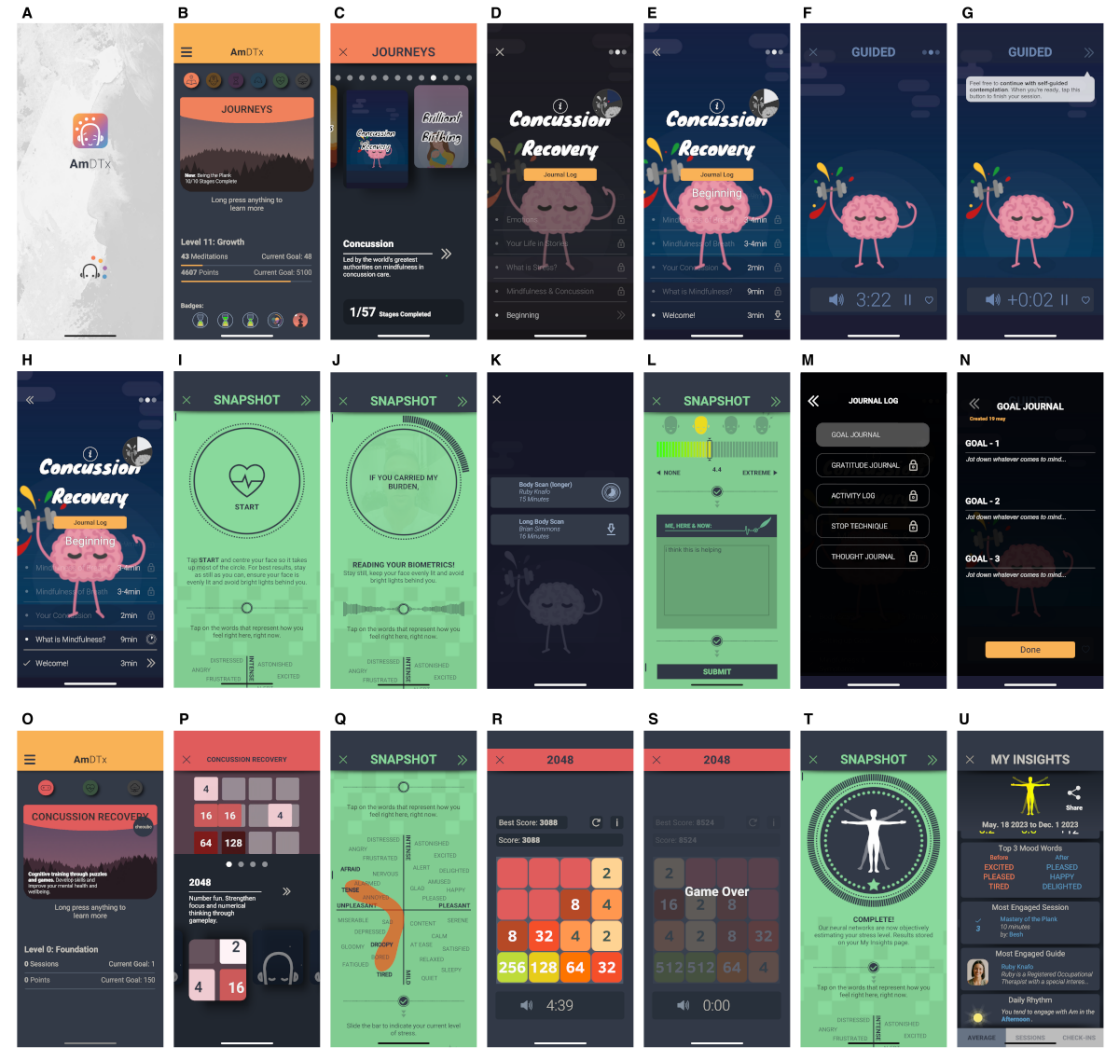
Adolescent concussion dedicated experimental and control interventions delivered through the AmDTx platform. (A) Initial loading screen of AmDTx; (B) main menu or home screen of AmDTx as seen by the participants randomized to the experimental intervention; (C) journey selection screen. Concussion Journey is shown; (D) main or menu page of the concussion MBI. Slices of the badge are awarded upon the completion of each module; (E) main or menu page of module 1; (F) stage 1 of module 1 play screen; (G) to complete the stage, the participant taps on the arrows at the top right; (H) main or menu page of module 3, with participant progression up to stage 5. (I, J and L) Before and after each the participant is prompted to complete the wellbeing measurements “Snapshot”; (K) stage 4 of module 2, “Body Scan” preplay screen with 2 meditation guide options; (L) snapshot ecological momentary assessment for stress and the open field box features; (M) journaling feature menu; (N) journaling feature input page relating to goal setting; (O) main menu or home screen of AmDTx as seen by the participants randomized to the control intervention. The feature “Concussion Recovery” is where the control math game is found; (P) main screen of the control task; (Q) to ensure as much similarity in the experience as possible and to collect valuable insights into the well-being of participants, the control is also book-ended with snapshots. Shown here is the mood circumplex feature; (R) control task midway through completion; (S) timer message prompting the participant to end their cognitive training for the day; (T) snapshot selfie scan feature after video has been captured; (U) the My Insights page where the participants randomized to either group can view how individual intervention stages or the control task impacted their well-being.

To be delivered via the *AmDTx* platform (Figure 2), the targeted DTx MBI training consists of a 4- to 8-week custom-made program (containing 8-modules) for adolescents with concussion, including setting intentions and check-ins on mood and stress, audio-recorded lectures, guided meditations such as focused attention exercises, and journaling events (Table 1). Each standardized psychoeducation or meditation practice will be unlocked as the participant progresses through the MBI program. Participants will be encouraged to participate in the DTx MBI activities for approximately 10 minutes every day, for a minimum of 4 days a week, over the 4- to-8-week period. Those who choose to end their therapy on the fourth week will be immediately directed to module 8 *Reviewing the program and how to maintain practice*. Those who choose to continue for an additional 4 weeks will move on to modules 5 through 8. The curriculum is based on previously validated *AmDTx* MBI app protocols [27,43] and team expertise in youth MBI (Sonia Roth, Craig Mackie, MC, and AAL) and was peer reviewed by experts in the field and parent engagement leaders (Ana Maria Vranceanu, Jonathan Greenberg, and Erin McCarthy). The intervention was piloted on 10 pediatric participants with concussion, and their feedback was integrated into the MBI and protocol [36]. We will track *meaningful* in-app use time, defined as the time spent engaging in meditation, psychoeducation, self-monitoring, and self-reflection, with a minimum threshold of 3 minutes per day. Any use time <3 minutes will not be counted toward the total weekly minutes spent using the *DTx.*

**Table 1:** Four- or 8-week experimental intervention consisting of a targeted digital therapeutics mindfulness-based intervention for adolescents with concussion that includes setting intentions and check-ins on mood and stress, audio-recorded lectures, guided meditations such as focused attention exercises, and journaling events.

Module	Course or focus	Meditation practices	Exercise
1	What is mindfulness Setting intentions Belly breathing exercise Introduction to body scan	Body scan (short): 7 minutes × 2 Belly breathing: 3 minutes × 2	N/A^a^
2	Mindful attitudes (nonjudgment and acceptance) How to skillfully meet concussion symptoms	Mindfulness of breath: 5 minutes × 2 Body scan: 15 minutes × 2	Setting goals Positive event journal Doing 1 daily routine mindfully
3	Stress response The biology of stress Inner narrative and chronic stress STOP technique	Mindful of breath: 10 minutes × 2 STOP technique Mindful movement: 7 minutes × 2	Symptoms of stress checklist and mapping stress on the body Journal when you used the STOP technique
4	Thoughts: the stories we tell ourselves Reframing symptoms with thoughts and attitudes Moving mindfully through your life	Mindfulness of sound transitioning to thoughts: 10 minutes × 2 Mindfulness movement: 10 minutes × 2	Thought journal
5	Pain and emotions How to mindfully manage pain Being mindful of your emotion The Breathe-Calm-Okay-Observe-Love technique	Body scan: 12 minutes × 2 Practice sitting meditation with emphasis on emotion: 10 minutes × 2	N/A
6	How to return to activities Gratitude—who or what has helped you in your recovery	Mindful movement practice: 10 minutes × 2 Loving-kindness and gratitude practice: 10 minutes × 2	Thought journal
7	Integrating mindfulness more fully into daily life Barriers to practice	Open awareness practice: 10 minutes × 2 Users choice practice: 5-15 minutes × 2	Thought journal
4 or 8	Review of program, strategies for maintaining practice, and reflection on how mindfulness has helped cope with your concussion or otherwise	Users choice practice: 5-15 minutes × 4	Review treatment goals Goal setting for the future

^a^N/A: not applicable.

#### Control Group Description: Sham App

To control for daily access to the *AmDTx* platform, expectation, and clinical attention bias, we created a sham DTx for the control group. The sham DTx will be delivered on the same main interface, within *AmDTx*; however, these participants will not have access to any of the MBI training or psychoeducational content. Their sham DTx will consist of playing an open-source math game called 2048 developed by Gabriele Cirulli [44]; this will be communicated to the control participants as *cognitive training*. The game requires participants to slide number tiles on a grid and combine them to create a tile with the number 2048. In a previous study, *AmDTx* was compared with 2048, revealing that 2048 did not reduce stress or improve psychological well-being in university students with anxiety [27]. Furthermore, 2048 was previously tested in a pediatric population with concussion, and the study found no evidence of harm or symptom improvement at 4 weeks [45]. It is expected that the control intervention will not bother individuals with sensitivity to light or high velocity motion because it lacks fast visuals or movement. The game is programmed to only be accessible for 10 minutes per day.

#### Coaching

All participants will be followed weekly by a coach through SMS text messaging. The coaches’ role will be to ensure that the participants adhere to the intervention. All the participants will receive weekly text messages from a coach. These messages will be sent on the same day and time every week and will be limited to 160 characters. The coaches will use a standardized question and answer protocol developed by the team. Coaches will also remind the participants to complete their follow-up surveys.

Furthermore, both interventions are equipped with automated reminders (triggered by inactivity).

#### Standard of Care

All the participants will receive standard of care [46] instructions pamphlet provided by RAs in the ED or at 360cc. The standard of care indicates that the patient refrains from physical and cognitive activities for 24 to 48 hours after injury. After the rest period, it is recommended that low-to-moderate levels of physical and cognitive activity be gradually started, including screen time. The activities should be performed at a level that does not result in the recurrence or exacerbation of symptoms. Adolescents must refrain from any activities that increase the risk of reinjury (eg, contact sports or anything with risk of falls) until fully asymptomatic and cleared by their primary care or other medical providers.

### Measures and Outcomes

#### Primary Outcomes

The prespecified thresholds (criteria for success), as outlined in Table 2 for the feasibility, are based on established benchmarks of previous feasibility trials [47,48] and our previous experience in recruiting pediatric patients with concussion from an ED [49]. We will use the *traffic light* system to determine whether proceeding to a full-scale trial is appropriate [50]. Progression can take place without any changes (green zone), with correctable adjustments (amber zone), or not occur because of issues that cannot be readily addressed (red zone). The decision to progress to a full-scale trial will be contingent on the criterion with the least favorable performance [50].

Table 3 provides an overview of all outcome measures collected and their specific end points.

**Table 2:** The criteria for success for progression to a full-scale efficacy trial.

Feasibility outcomes	Green zone (go)	Amber zone (amend)	Red zone (stop)
Eligibility	>40% of participants screened are eligible	20%-40% of participants screened are eligible	<20% of participants screened are eligible
Recruitment	>50% of eligible participants will be randomized [49]	30%-50% of eligible participants will be randomized	<30% of eligible participants will be randomized
Adherence	>70% of participants complete the minimum requirements, that is, approximately 10 minutes of activity 4 times a week	>50% of participants complete the minimum requirements, that is, approximately 10 minutes of activity 4 times a week	<50% of participants complete the minimum requirements, that is, approximately 10 minutes of activity 4 times a week
Credibility^a^	>70% of participants have score above the scale midpoint	>50% of participant have a score above the scale midpoint	<50% of participants have a score above the scale midpoint
Retention	>75% participants complete the follow-up assessment [49]	50%-75% participants complete the follow-up assessment	<50% participants complete the follow-up assessment

^a^Treatment credibility and expectancy will be assessed using the Credibility and Expectancy Questionnaire [51]. It is a 6-item questionnaire, scored using a Likert scale ranging from 1 to 9. The treatment will be considered credible (questions 1-3) if the participants scored above the midpoint. Furthermore, credibility and expectancy will be assessed to rule out the possibility of differential credibility as an explanation for the differences in therapy outcomes between the experimental and control groups. The credibility and expectancy of the intervention will be assessed at 1 week after enrollment.

**Table 3:** Measures organized by domain, source of data, and assessment occasion.

Measures				Source or reporter		Time to complete (minutes)		CHEO ED^a^		360cc^b^		Daily^c^		Day 1 (CHEO)		Day 1 (360cc)		72-hours		Day 7		Day 14		Week 3		Week 4		Week 8
**ED visit**																												
	Screening form			RA^d^		N/A^e^		✓		✓																		
	Case report (acute signs and symptoms)			RA and MD^f^		5 RA and 5 MD		✓		✓																		
	Balance (balance error scoring system)			CP^g^		5-10		✓		✓																		
**Outcomes**																												
	**Psychological**																											
		Self-Efficacy Questionnaire for Children	C		2-5								✓		✓										✓		✓	
		Connor-Davidson Resilience Scale	C		2-5								✓		✓										✓		✓	
		Generalized Anxiety Disorder 7-item scale	C		2-5		✓								✓										✓		✓	
		Center for Epidemiologic Studies Depression Scale for Children	C		2-5		✓								✓										✓		✓	
		Child and Adolescent Mindfulness Scale	C		2-5		✓								✓										✓		✓	
		Credibility Expectancy Questionnaire	C		5														✓									
		Client Satisfaction Questionnaire	CP		5																				✓			
	**Postconcussive symptoms (secondary)**																											
		Health and Behavior Inventory	C		5-10		✓		✓												✓				✓		✓	
		Post-Concussion Symptom Inventory	C		5		✓		✓												✓				✓		✓	
	**Functional impairments (secondary)**																											
		Quality of Life (PedsQL^h^)^i^ scale	C		5-10		✓								✓										✓		✓	
		PedsQL Fatigue scale	N/A		N/A																				✓		✓	
	**Neurophysiological measures (subset of 60 participants)**					45																						
		Arterial spin labeling	C														✓								✓			
		Resting-state fMRI^j^	C														✓								✓			
		MRS^k^	C														✓								✓			
**Cognitive measures**																												
		NIH^l^ Toolbox	C		25																				✓			
		App Measurements	N/A		N/A						✓																	
		Godin-Shephard Leisure-Time Physical Activity Questionnaires	C		5								✓		✓						✓				✓		✓	
		Puberty Questionnaire	C		5		✓								✓													

^a^CHEO ED: Children’s Hospital of Eastern Ontario emergency department.

^b^360cc: 360 Concussion Care.

^c^When accessing the app.

^d^RA: research assistant.

^e^N/A: not applicable.

^f^MD: treated ED physician.

^g^CP: concussed child and parent or guardian.

^h^PedsQL: Pediatric Quality of Life Inventory.

^i^Outcome measure.

^j^fMRI: functional magnetic resonance imaging.

^k^MRS: magnetic resonance spectroscopy.

^l^NIH: National Institutes of Health.

#### Secondary Outcomes

##### Treatment Expectancy

*Treatment expectancy* will be assessed with the Credibility and Expectancy Questionnaire (questions 4 to 6) [51]. Expectancy will be considered good if the participants score above the midpoint on questions 4 to 6. Expectancy will be assessed at 1 week after the enrollment.

##### Intervention Satisfaction

*Intervention satisfaction* will be assessed with the modified version of the Client Satisfaction Questionnaire [52]. This is a validated, reliable, 8-item questionnaire used to measure client satisfaction with a particular program or service. In this case, the questionnaire has been modified to assess participant satisfaction with the digital intervention. The intervention will be deemed satisfactory if >70% of the participants score above the midpoint on the questionnaire [52]. Intervention satisfaction will be assessed at 4 weeks after the enrollment (end of the intervention).

##### Quality of Life

*QoL* will be measured using the Pediatric Quality of Life Inventory (PedsQL; version 4.0) [53,54]. The PedsQL is a reliable and valid measure of QoL in healthy children and adolescents and in those with acute or chronic health conditions [55]. It is a 23-item, 5-point Likert scale questionnaire yielding a total score (0-92) and 4 domain scores: physical, emotional, social, and school. QoL will be collected in the ED (for CHEO participants) or on day 1 (for 360cc participants) and at the 4- and 8-week follow-ups.

##### Fatigue

*Fatigue* will be measured using the Multidimensional Fatigue Scale PedsQL. This 18-item, 5-point Likert scale assessment comprises 3 scales: the General Fatigue Scale (6 items), Sleep-Rest Fatigue Scale (6 items), and Cognitive Fatigue Scale (6 items). The PedsQL Multidimensional Fatigue Scale has demonstrated excellent internal consistency, reliability, and validity [56-58]. The assessment will be collected in the ED (for CHEO participants) or on day 1 (for 360cc participants) and at the 4- and 8-week follow-ups.

##### Symptom Burden

*Symptom burden* will be measured in the ED or at 360cc (both retrospective and postinjury acute symptoms) and at 1, 2, 4, and 8 weeks after enrollment using the Health and Behavior Inventory (HBI) [3]. The HBI is a 20-item self-report questionnaire that uses a 4-point Likert scale, with a total range 0-60. It provides scores for cognitive and somatic symptom scales. Emotional symptoms and sleep disturbances will be assessed using the Post-Concussion Symptom Inventory (PCSI) [59-61]. The PCSI [59,60] is a validated, reliable, comprehensive, and self-administered instrument [59-61]. For the purpose of this study, the emotional and sleep domains of the PCSI adolescent scale version (20-item, 7-point Likert scale) will be used. The domains will be assessed in the ED or at 360cc and at 2, 4, and 8 weeks after enrollment.

##### Self-Efficacy

*Self-efficacy* will be measured using the Self-Efficacy Questionnaire for Children (SEQ-C). Self-efficacy is the individual’s perceived and personal competence in their abilities to handle a situation. Self-efficacy reflects one’s confidence in the ability to exert control over one’s motivation, behavior, and social environment. Previous studies have demonstrated that at 12 weeks after the injury, children with a concussion lack confidence during physical activities at 12 weeks after a concussion as compared with that before the injury [62]. The SEQ-C is a valid and reliable assessment tool. It is a 24-item, 5-point Likert scale yielding a sum score (0-120) and emotional, social, and academic self-efficacy subscores [63]. The SEQ-C will be administered on day 1 and at 4 and 8 weeks after enrollment.

##### Attention, Working Memory, and Executive Function

Attention, working memory, and executive function will be assessed using the National Institutes of Health (NIH) Toolbox Cognitive Battery [64]. The NIH Toolbox is a validated and reliable computerized battery designed to measure executive function, attention, episodic memory, language, processing speed, working memory, and fluid cognition. Previous studies have demonstrated lower working memory, attention, and executive functioning scores after injury in pediatric samples with concussion [65-68]. Moreover, mindfulness has been shown to improve attention, working memory, and executive functioning [26,69,70]. The NIH Toolbox will be administered at the 4-week follow-up.

##### Resiliency

*Resiliency* will be measured using the Connor-Davidson Resilience Scale-10 (CD-RISC-10) [71]. The CD-RISC-10 is a 10-item questionnaire, scored on a 5-point Likert scale, that assesses an individual’s perception of hardiness or perceived stress. Resiliency is defined as the ability to harbor interpersonal qualities, such as positive acceptance, to adapt or thrive in the face of adversity [71]. Previous research has shown that MBI increases resiliency in youth [13]. The CD-RISC-10 has been validated in an adolescent population with a concussion [72] and will be administered on day 1 and at 4 and 8 weeks after enrollment.

##### Anxiety and Depression

*Anxiety* and *depression* (mood) are frequently reported symptoms in pediatric concussion [73-75]. *Anxiety* will be measured using the Generalized Anxiety Disorder 7-item scale [73]. The Generalized Anxiety Disorder-7 is a validated, reliable, and sensitive to treatment-related changes tool that assesses anxiety symptoms in youth [76]. It is a 7-item, 3-point Likert scale that provides a sum score (0 to 21) of general anxiety symptoms. It will be administered in the ED (for CHEO participants) or on day 1 (for 360cc participants), capturing preinjury anxiety, and at 4- and 8-week intervals after enrollment to assess postenrollment anxiety.

*Depression* symptoms will be measured using the validated and reliable Center for Epidemiologic Studies Depression Scale for Children [74,75]. It is a 10-item, 4-point Likert scale that provides a total score (0-30). The Center for Epidemiologic Studies Depression Scale for Children will be administered in the ED (for CHEO participants) or on day 1 (for 360cc participants), capturing preinjury mood, and at 4- and 8-week intervals after enrollment to assess postenrollment mood.

##### Mindfulness

*Mindfulness* will be assessed using the validated Child and Adolescent Mindfulness Measure [77,78]. It is a 10-item, 5-point Likert scale. It will be administered in the ED (for CHEO participants) or on day 1 (for 360cc participants), capturing preintervention mindfulness, and at 4- and 8-week intervals after enrollment to assess postenrollment mindfulness.

##### Safety

*Safety* of the MBI will be determined by capturing and monitoring the worsening of symptoms and adverse events. Adverse events will be defined as an unscheduled visit to the ED or primary medical provider because of exacerbation of symptoms during or within 30 minutes after using the app. The participant will be asked about any possible adverse events at the 1-, 2-, 4-, and 8-week follow-ups.

##### Longitudinal Mental Well-Being

*AmDTx* contains 4 easy-to-use measurement features that together provide a longitudinal readout of patient well-being. First, psychological stress is objectively quantified via a 30-second *selfie* video that uses photoplethysmography to extract heart rate and 2 power bands of heart rate variability from biosignals inherent to the human face [79]. The amount of psychological stress is determined via a deep neural network trained on tens of thousands of selfie scans captured in parallel with a validated ecological momentary assessment of stress. The artificial intelligence output has reported 86% accuracy for determining an individual’s stress as *very low*, *medium low*, *medium high*, or *very high* [80]. Second, emotional affect and arousal levels are obtained via a digital 4-quadrant emotion mapping board (circumplex) that lists feelings such as *afraid*, *droopy*, and at ease ranging on one axis from unpleasant to pleasant (valence axis) and along another axis from mild to intense (arousal axis). Each emotion listed is associated with a score that is not disclosed to the user and is used to calculate an implicit measure of mood. Third, subjective stress is obtained via a slider ranging from *none* to *extreme*. Fourth, personal notes are imputed using an open field text box. The outputs of the mood and stress slider ecological momentary assessments have been benchmarked to standard psychological surveys [27]. If a participant chooses to use the selfie camera to observe their biological stress levels, there will be no recording or storage of the generated video in the app or elsewhere. The AmDTx uses secure web authentication, data logging, and encryption that ensure security and confidentiality of any personal identifiable information and in-app data. Personally identifiable information remains with the customer (ie, it is not downloaded by Mobio).

##### Imaging Measurements

*Imaging measurements* will be acquired within 72 hours of injury and during week 4 of the study on a subset of 60 participants. Each brain scanning session lasts 45 to 60 minutes and includes the following sequences: 3D T1-weighted anatomical scan to measure regional volumes and cortical thickness, resting-state functional MRI (rs-fMRI) to assess functional connectivity features or neural networks, arterial spin labeling to assess perfusion or cerebral blood flow, and magnetic proton spectroscopy in a single voxel in the anterior cingulate cortex to assess metabolite concentrations (ie, glutamate and gamma-aminobutyric acid). Table 3 lists the key imaging parameters.

#### Covariate Measurements

Information about injuries will be collected from medical records and medical personnel using a standardized case report form. Details regarding the injury and acute signs and symptoms of concussion (ie, loss of consciousness, Glasgow Coma Scale scores, mechanism of injury, neurological status, and other clinical features) will be collected by RAs, with clarification by physicians when necessary. The data will be verified by the site investigator. To calculate the 5P clinical risk score [2], balance will be evaluated by the RA using the balance error scoring system [81], a widely used instrument in the field of sports concussion [82] for objective assessment of postural stability. Pubertal status will be measured using a puberty questionnaire [83]. The questionnaire is a 5-item, self-administered rating scale for pubertal development without pictorial representations or interviews. Demographic data (ie, household annual income and parental education level) will be used as a proxy of socioeconomic status (SES). SES will be collected to assess whether randomization is equally distributed in both groups. If SES is not equally represented in the sample, it will be controlled for in our analysis.

Studies have shown that physical activity can reduce anxiety, improve mood, and help alleviate postconcussive symptoms [84]. We will evaluate physical activity to ensure balance between groups; physical activity levels will be assessed using the Godin-Shepard Leisure-Time Physical Activity Questionnaire [85] on day 1 and then at 2, 4, and 8 weeks after injury.

### Procedures

#### Data Collection Methods

Recruitment will be conducted similar to the successful Pediatric Concussion Assessment of Rest and Exertion multicenter concussion study [86]. When an adolescent with a mechanism of injury and symptoms consistent with concussion presents to the ED, the nurse will triage the child as per the existing protocols. Once in the ED, a member of the ED clinical team (ie, an ED volunteer) will introduce the study to families of potentially eligible children to inquire whether they are interested in learning more about the study. If so, the electronic charts of potentially eligible participants will be flagged, and the research team will be notified. At 360cc, a health information custodian who is a part of the circle of care will mention to the family that there is a study on children who have head injuries and ask if they are interested in learning more about the study. In both the ED and 360cc, the RA will then approach the family to discuss the research study, and if they are interested, the RA will complete the screening form on an iPad.

During the screening procedure, we will evaluate the likelihood that the patients had a *definitive* or *probable* concussion based on the aforementioned inclusion criteria. Their 5P clinical risk score [2] will also be calculated with the following variables: age, sex, prior concussion and length of recovery, history of migraine, slow answers to questions, balance, headache, sensitivity to noise, and feeling fatigued. Only patients with a 5P score >4 will be eligible for the study. Patients who are ineligible based on the initial screening will be thanked for their time. Eligible and willing parents along with adolescents capable of consenting on their own behalf will be asked for written informed consent, and the adolescents who are unable to provide consent on their own behalf will be asked for assent.

Once enrolled in the study, the families will complete a series of questionnaires in an electronic survey format using a portable tablet computer on REDCap [87]. The survey includes questions regarding patient demographics, preinjury and injury characteristics, a symptoms checklist, and diagnostic history.

The day following the ED or 360cc visit, participants will receive a phone call from an RA to randomize the participants to either the experimental or the control group and give instructions to register an account on a study-dedicated landing page using a unique link for each participant. Following registration, participants will install *AmDTx* on the mobile device of their choice. After creating an account and logging in to *AmDTx*, each participant will be automatically directed to their assigned intervention (MBI or control).

During this phone call, an MRI appointment will be scheduled for the participants who have agreed to participate in that component of the study. Participants will complete the surveys or questionnaires for day 1 and MRI screener. They will have the option of completing the survey via phone or REDCap.

#### Follow-Up Procedures

The proposed duration of the app-based MBI is 4 to 8 weeks. After 4 weeks, all participants in the experimental group will be given the opportunity to pursue the MBI for an additional 4 weeks. At that time, participants in the control group will be offered to cross over and start the MBI intervention.

Participants will complete electronic follow-up questionnaires on REDCap at 1, 2, 4, and 8 weeks after enrollment and will have an in-person follow-up at 4 weeks for the cognitive assessment (and the MRI scan for a subset of participants). Cognitive testing will be conducted before brain scanning.

#### Neuroimaging Procedures

A subset of 60 participants (30 from each group) will undergo a 45-minute MRI scan at <72 hours and 4 weeks after injury. During the day 1 phone call, participants who have consented to the MRI component of the study will complete the MRI screener and schedule their MRI appointment.

Each scanning session will be approximately 1 hour and will include a high-resolution structural scan, arterial spin labeling, rs-fMRI, and magnetic proton spectroscopy (Table 4).

**Table 4:** Magnetic resonance imaging acquisition.

Image type	Sequence name	Total time (minutes:seconds)	Resolution (mm×mm×mm)	Slices, n	FOV^a^ (mm)	TR^b^ (milliseconds)	TE^c^ (milliseconds)	Other
High-resolution anatomical	MEMPRAGE	5:48	1.0**×**1.0**×**1.0	192	256	2500	1.69, 3.55, 5.41, and 7.27	Flip angle=7; inversion time=1050 milliseconds; phase FOV=100%
rs-fMRI^d^	—^e^	12:08	3.0**×**3.0**×**3.0	40	204	2160	25	Flip angle=70; acceleration factor=2; 334 volumes
ASL^f^	3D ASL (GRACE)	5:37	1.8**×**1.8**×**5.0	34	230	4600	15.56	Perfusion mode=Flow-sensitive Alternating Inversion Recovery-QII; bolus duration=700 milliseconds; inversion time=860 milliseconds, 1060 milliseconds, 1260 milliseconds, 1460 milliseconds, 1660 milliseconds, 1860 milliseconds, 2060 milliseconds, 2260 milliseconds, 2460 milliseconds, 2660 milliseconds, 2860 milliseconds, 3060 milliseconds; average=1
ASL	Calibration image (M0)	0:54	1.8**×**1.8**×**5.0	34	230	7500	15.92	Perfusion mode=Flow-sensitive Alternating Inversion Recovery-QII; bolus duration=700; inversion time=7000 milliseconds; average=1
MRS^g^	PRESS	4:38	40**×**20**×**15	—	—	2000	30	Average=128
MRS	MEGAPRESS	4:36 (×2)	40**×**20**×**15	—	—	2000	68	Average=64

^a^FOV: field of view.

^b^TR: repetition time.

^c^TE: echo time.

^d^rs-fMRI: resting-state functional magnetic resonance imaging.

^e^Not available.

^f^ASL: arterial spin labeling.

^g^MRS: magnetic resonance spectroscopy.

#### Data Management

The CHEO Clinical Research Unit Data Coordinating Centre will be used as a central location for data processing and management. Data will be kept private and secured to industry standards for both clinical and patient-sensitive data in Canada. Data for the study will be collected and managed using REDCap [88] tools hosted and supported by the CHEO Research Institute. REDCap is a secure, web-based application designed to support data capture for research studies. Only members of the research team will be granted access to the database. Users will be assigned to *Data Access Groups* that will restrict their rights to viewing and entering data. For monitoring purposes, the study coordinator will be able to view the data. Within the Data Access Group, user privileges will be designated by the study coordinator to ensure that the research team members have only the minimum required rights to perform their duties. All identifying information that is collected will be flagged in the database and removed from data export unless the identifying information is required for statistical analysis (eg, date of birth).

### Statistical Analysis

#### Primary Outcomes

Descriptive statistics will be used to summarize patient baseline characteristics and clinical information. The primary objective of this study is to assess the feasibility outcomes of the trial. This will be done by computing the observed points estimates for each criterion (eligibility, recruitment, adherence, credibility, and retention) and comparing them with our predefined red zone upper limits and green zone lower limits. The 3-tiered progression criteria will be used for each criterion (Textbox 1).

**Three-tiered progression criteria where E denotes the observed point estimate (ranging from 0 to 1 for proportions or percentages 0%-100%) [50].**

**Progression criteria**

- Red (unacceptable): E≤red zone upper limit (*P* value nonsignificant, *P* value≥α)
- Amber (amend): red zone upper limit<E<green lower limit
- Green (acceptable): E≥G lower limit (*P* value significant, *P* value<α)

#### Secondary Outcomes

Mean and SDs will be computed for each collected measure. Pre-post correlations will be generated to assess preliminary evidence of *efficacy signal* for QoL, fatigue, symptoms, resiliency, self-efficacy, cognition, anxiety, depression, and mindfulness.

#### Neuroimaging Component: Nested Study

To acquire preliminary data on the association between the interventions and MRI metrics, per-protocol analyses will be conducted. A multivariable linear regression will be performed for each neuroimaging outcome, with age, sex, and handedness (for rs-fMRI) as covariates. If the groups differ at baseline on potentially confounding variables, such as attention-deficit or hyperactivity disorder, anxiety, medication, prior concussion, physical activity, and puberty, these variables will also be entered as covariates in the models. Per-protocol analyses will include only those participants who have adhered to the intervention.

#### Statistical Power

##### Feasibility Objective

The sample size calculations were informed on recommendations for feasibility trials using the *traffic light* system [50,89]. The significance cutoff points for each criterion, with green lower and red upper limits, were determined. Using Table 1 from the study by Lewis et al [50], to meet the individual >90% power requirements for each of the five criteria we would need the following: (1) for eligibility, the number of participants to be screened is 46; (2) for recruitment, the number of participants to be randomized is 54; (3) for adherence, the required number of participants to evaluate adherence is 55; (4) for credibility, the required number of participants is 55; and (5) for retention, the required number of participants is 34. To determine the overall sample size for the whole study, we based our decision on the criterion that requires the largest numbers (ie, adherence, n=55). Considering an approximately 20% rate of loss to follow-up and withdrawals, the required sample size for the adherence criteria is 70. We expect that 40% of the participants screened will be eligible and there will be a 50% recruitment uptake. Given these numbers, 350 children are expected to be screened: 350=[(1/0.4)×(1/0.5×70)]. Assuming that 70 of the 350 screened participants are randomized, criteria 1, 2, 3, 4, and 5 will have statistical powers of 100%, 99.9%, 95.8%, 95.8%, and 98.7%, respectively. This means that the null hypotheses can be rejected with a collective power of 90.5% if the alternative hypotheses (for acceptable feasibility outcomes) are true in each case.

##### Sample Size for the Nested Neuroimaging Component

As per Desmond and Glover [90], a liberal threshold of α=.05 requires 24 participants (12 per group) to achieve an 80% power for the single-voxel level for typical activations. To control the error rate when conducting multiple comparisons, it is suggested that the number of participants should be doubled to maintain this level of power. On the basis of our Pediatric Concussion Assessment of Rest and Exertion and MRI study, of the 89 participants with concussion who provided consent, 13 were lost to follow-up, 3 had incidental findings, and 5 had excessive motion [91]. Considering a 20% attrition, a total sample size of 60 participants (30 per group) will be recruited.

### Data Safety and Monitoring

#### Data Safety Monitoring Board

The independent data safety monitoring board (DSMB) will consist of 1 independent pediatric ED physician, 1 neuropsychologist who specializes in pediatric concussion, and 1 statistician who is not involved in the research study. In keeping with the StaRChild Health guidelines [92], the DSMB will, in collaboration with the trial steering committee, establish safety outcomes and termination rules before the start of the study. The DSMB will be immediately advised of severe adverse events, and they will meet in case a serious adverse event occurs. The DSMB will meet annually to review enrollment, study procedures, form completion, data quality, loss to follow-up, and interim safety results. Given the low-risk intervention and no intention to stop the trial for benefit or futility, only an annual meeting will be required. On the basis of negative data trends (increased symptoms) and adverse events, the DSMB may decide to meet earlier than planned and may request unblinding if deemed necessary.

#### Potential Harms

The safety outcomes include (1) severe worsening of symptoms identified on HBI reliable changes or (2) any symptoms requiring an unscheduled visit to the ED or primary care provider. If concussion symptoms worsen during the treatment to the degree that the participant requires an unscheduled visit to the ED or primary care provider, then symptoms will be considered as a possible adverse event. Symptoms could develop into an adverse event if they become unbearable and the patient needs immediate medical attention. Patients will report possible adverse events through REDCap reports, and their responses will be monitored by RAs. In the case of a possible adverse event, defined as the *worsening of symptoms requiring an unscheduled visit to the ED or primary care provider*, site investigators will be notified to determine if the event is linked to the treatment. If linked, the event will be monitored and the CHEO Research Ethics Board and DSMB will be notified.

### Ethical Considerations

This study was approved by the Research Ethics Board (protocol number 20/72X) of the CHEO Research Institute in August 2020. Authorization from Health Canada was granted in July 2022. The study will be carried out according to the principles outlined in the Declaration of Helsinki [93] and Good Clinical Practices, within the laws and regulations of the Tri-Council Policy Statement, and the institutional policies of the CHEO REB.

Before consent is sought, the study will be introduced, and its purpose will be explained verbally. The consent form will also present the purpose of the research, the procedures, potential risks and benefits, and the use and security of the data. Informed written consent will be obtained from the parents and participants deemed capable of consenting. Informed assent will be obtained from the participants deemed by the RA to be cognitively unable to provide informed consent.

The participants will be remunerated with a CAD $20 (US $15) gift certificate (eg, Tango) for completing surveys. Participants will receive an additional CAD $20 (US $15) gift certificate for completing the MRIs. The gift certificate will be sent electronically to their email address. They will also receive a parking voucher or vouchers for in-person meeting or meetings and a letter attesting that they have completed 20 hours of volunteer work (5 hours per week completing the intervention).

Participant information will be coded using study identification numbers. Research personnel will take all appropriate and customary steps to ensure that data remain secure and that patient privacy and confidentiality are maintained.

## Results

### Project Initiation

This trial is funded by the Children’s Hospital Academic Medical Organization Innovation Fund and the multidisciplinary team grant from the University of Ottawa Brain and Mind Research Institute. Recruitment began in October 2022, and data collection is expected to be completed by September 2024. Data collection and management is still in progress; therefore, data analysis is yet to be conducted.

### Dissemination

The results will be disseminated at international conferences and in scientific manuscripts to peer-reviewed journals. The main manuscript presenting the results of the primary objective will be published in a high-impact peer-reviewed journal. Furthermore, separate manuscripts will be written on the secondary objectives of the protocol, and these will also be submitted for publication in peer-reviewed journals. We plan to engage key stakeholder groups (eg, families, Parachute, Canadian Concussion Network, and Mobio Interactive) to ensure mobilization and uptake of the findings to end users to progress to the next step, that is, the efficacy trial.

## Discussion

First, we will use this study to examine the feasibility of our RCT methods, including delivering a DTx-containing MBI to participants who are acutely or subacutely concussed, and whether we can move to the efficacy trial. Second, to better inform our larger RCT, this study will allow us to estimate the effect sizes of QoL and the symptoms at 4 and 8 weeks after enrollment compared with those randomized to the control group (sham intervention). Third, we will use this study to assess whether MBI induces measurable brain-based effects, that is, examine whether the intervention modulates neuroimaging indicators such as functional connectivity. This trial will offer insight into a cost-effective way to manage pediatric concussion in the acute and subacute phases of injury. This study will help support the development of a future full-efficacy trial to assess the effectiveness of the intervention in increasing QoL and decreasing the concussion symptom burden.

The development of an intervention to increase adaptive interpersonal qualities in acute settings is important for reducing the risk of PSAC. Formal in-person MBIs such as mindfulness-based stress reduction have been shown to increase perceived self-efficacy and teach positive acceptance of change [13]. However, these interventions may not be suitable for a youth population with acute concussion as they are costly and not easily accessible. A brief web-based MBI with guided meditation had positive effects on anxiety, negative mood, perceived stress, and attention in different clinical populations [24-27]. Furthermore, a 3-week RCT using the DTx MBI within *AmDTx* increased stress resilience and attentional control in university students self-described as experiencing mild anxiety [27]. The customization of a novel MBI, designed in collaboration with MBI therapists, patients, and Mobio Interactive, will bring a required concussion treatment into the homes of all patients, with the goal of increasing adaptive skills and cognitive performance, promoting neural recovery, and reducing the risk of PSAC. If found to be effective, DTx MBI holds promise as an accessible and low-risk preventative treatment for youth at risk for prolonged postconcussion symptoms and sequelae.

## Appendix

Multimedia Appendix 1 Peer review comments from the Children's Hospital Academic Medical Organization (CHAMO), Innovation Fund.

Multimedia Appendix 2 Peer review comments from the University of Ottawa Brain and Mind Research Institute Team Grant.

Multimedia Appendix 3 Peer review comments from the Children's Hospital of Eastern Ontario Research Insitute Research Growth Award.

## Abbreviations

360cc: 360 Concussion Care
5P: Predicting and Preventing Post-Concussive Problems in Pediatrics
CD-RISC-10: Connor-Davidson Resilience Scale-10
CHEO: Children’s Hospital of Eastern Ontario
DSMB: data safety monitoring board
DTx: digital therapeutics
ED: emergency department
HBI: Health and Behavior Inventory
MBI: mindfulness-based intervention
MRI: magnetic resonance imaging
NIH: National Institutes of Health
PCSI: Post-Concussion Symptom Inventory
PedsQL: Pediatric Quality of Life Inventory
PSAC: persisting symptoms after concussion
QoL: quality of life
RA: research assistant
RCT: randomized controlled trial
REDCap: Research Electronic Data Capture
rs-fMRI: resting-state functional magnetic resonance imaging
SEQ-C: Self-Efficacy Questionnaire for Children
SES: socioeconomic status
TBI: traumatic brain injury

## Data Availability

Data sharing is not applicable to this paper as no data sets were generated or analyzed during this study.
