# Supplementary material for: Smartphone App–Delivered Mindfulness-Based Intervention for Mild Traumatic Brain Injury in Adolescents: Protocol for a Feasibility Randomized Controlled Trial
Source: JMIR Res Protoc. 2024 Apr 11;13:e57226. doi: 10.2196/57226 (PMC11046387; doi:10.2196/57226)
Supplement: Multimedia Appendix 1 [file resprot_v13i1e57226_app1.pdf]

Principal Investigator: Roger Zemek
Title: Mindfulness-Based Intervention for Mild Traumatic Brain Injury (MBI-4-mTBI)

REVIEWER 1 COMMENTS

Team strengths
Please comment on (as appropriate): This team comprises experts with experience in the specific components of the protocol to which they are assigned. The app developer is experienced and has much work already done (e.g., technical aspects, methods to secure privacy, etc.) and is donating his time and effort. The researchers have good track records of completing studies and disseminating the knowledge. Successful completion of the project and sharing the knowledge to the identified populations is highly likely with this team.

Proposal Merit
The proposal addresses an important clinical question based on clinical neuroscience. It is feasible to answer and the hypothesis is sound. An appropriate design has been chosen to answer the question and test the hypothesis. A large enough sample will be recruited and the methods of recruitment have been successfully used before and are likely to yield enough Ss within the time frame, although the study is quite ambitious for two years. The questionnaires selected are appropriate for the topic, constructs, and age groups. The intervention will be developed by an expert in the field. Statistical analyses are appropriate, but simplistic, e.g. there is no intention to treat analysis planned. The impact could be high if successful and widely disseminated, both of which are likely to happen.

Innovation
The purpose of the CHAMO Innovation Awards is to *" ...to support the development of new and innovative approaches to health care delivery and to provide leadership in the dissemination of new knowledge across the healthcare system. These projects will no doubt impact healthcare delivery and offer opportunities to translate new knowledge into medical practice locally and nationally."*
This proposal is innovative in 3 ways:

1. It uses wellness intervention of the mind to address a widespread, serious health problem in children and adolescents. This is an unusual problem-intervention combination.
2. The study will develop an app to make the intervention more portable and accessible, thus enhancing health service delivery.
3. The app will be freely disseminated if it works, which will further enhance health care delivery in a novel manner.

Assessment
The instruments and protocol used to measure treatment response are appropriate and sound. The statistical analysis is simplistic, but appropriate. The timeline is reasonable, although it is an ambitious study that could not be carried out without support from other sources. However, those sources have already been secured.

Potential impact
Impact depends on successful research and a plan to promote and disseminate the work. This study, if the intervention is successful, will have a large impact on the population of patients with pediatric TBI. There is sound scientific reasoning underlying the hypothesis which makes the chances of success high. The knowledge translation and sharing of the method are well-planned and feasible.

REVIEWER 2 COMMENTS

Team strengths
Strong team

Proposal Merit
Excellent writing and proposal, however, I do have questions about starting with an app:

- It requires a lot of resources to create app for the iOS and Android ecosystem.
- Unless resources are available for ongoing development, the app will rapidly become out of date.

Instead of a mobile app however, I might recommend creating a mobile first website, a website with flexible, liquid, dynamic layout that can be viewed regardless of platform.

Advantages of a mobile-first website:

- It is available on: 1) on a desktop, 2) tablet, or 3) smart phone.
- Fewer resources to maintain.

On the other hand, I appreciate that perhaps that there is additional app-based data you want to gather that you cannot from a website.

E.g. location tracking for analysis of user's daily routines, etc.
E.g. tracking screen time usage of the devices, etc.

If your project is successful, then over time, you can "port" your website into app versions for iOS/Android.

Innovation
It is innovative that Zemek has:

- Taken mindfulness (which has excellent evidence-based for various brain conditions, such as mental health and stress), and wondered if it might be helpful for concussions.
- Considered using a mobile app which will increase deliverability of the intervention.

I am curious to see how recommending an app (i.e. recommending screen time) will affect people with concussions.

Assessment
Metrics appear sound.

Potential impact
The research question is a very important and relevant question
