# Supplementary material for: Smartphone App–Delivered Mindfulness-Based Intervention for Mild Traumatic Brain Injury in Adolescents: Protocol for a Feasibility Randomized Controlled Trial
Source: JMIR Res Protoc. 2024 Apr 11;13:e57226. doi: 10.2196/57226 (PMC11046387; doi:10.2196/57226)
Supplement: Multimedia Appendix 3 [file resprot_v13i1e57226_app3.pdf]

**Principal Investigator:**	Andree-Anne Ledoux
**Title:**	Mindfulness-Based Intervention for Mild Traumatic Brain Injury in youth with MRI (MBI-4-mTBI+MRI)
**Score:**	4.08
**Rank:**	3 out of 8

SCIENTIFIC OFFICER NOTES

This was a very strong application: well-written, clearly defined objectives, realistic workplan, and a strong team behind this new applicant. As an add-on study to a well-supported project it will overcome many barriers to study start up and benefits from significant cofounding from CHAMO parent study and company. The adequacy and clarity of the sample size was discussed, but it is recognized that a larger sample size is likely not feasible. Cited evidence is for adults, and although childhood brain development is very different this was not addressed in the proposal. Applicability of apps in children is also not discussed. Family leader feedback was incorporated, particularly social media strategy for dissemination and parents will be able to track mindfulness activities during the four week period.

REVIEWER COMMENTS

Review 1

The applicants propose a neuroimaging study is to 1) establish neurophysiological evidence for the effectiveness of an early app-based Mindfulness-based intervention delivered through an app, using objective MRI outcome measures of recovery and 2) examine the association between changes in neurophysiological DMN (disruption of the default-mode network) indices and clinical and neuropsychological measures in concussed youth at 4-weeks. This is not an independent study, but an adjunct pilot study to a larger CHAMO-funded trial in which children aged 10-18 years with acute concussion are randomized to early MBI or usual care (30 out of 275 children).

Strengths: There is significant co-funding from the parental study (CHAMO) and via getting to use the app/services without license fees (Mobio). As part of the larger study many barriers to feasibility (ethics, recruitment, etc) are already addressed and the scientific value and impact have already been evaluated as excellent.

Weakness: I am not sure whether the imaging results will be consistent across a relatively small subset of study subjects, especially if high variability is expected (power calculation?), but some important pointers towards the usefulness of the imaging outcome measures and their correlation with neuropsychology may be derived. While the imaging component is new and could be considered as a pilot, the whole study is within the frame of previous and ongoing work of the investigator.

Review 2

Andrée-Anne Ledoux et al are presenting a study proposal to assess the effect of an intervention with a mindfulness-based app on post-concussion symptoms and their persistence through functional MRI exams.

The rationale for the study is clear as self-reported outcomes to interventions may be less reliable than functional MRI assessments which may be more objective.

The proposal is very well written with clear definitions of objectives, reliable sample size calculation, clear description of methodology, analytical methods and a realistic work plan.

The topic is very relevant with a large number of youth affected by post-concussion symptoms with potential long term negative effects.
