# Supplementary material for: Smartphone App–Delivered Mindfulness-Based Intervention for Mild Traumatic Brain Injury in Adolescents: Protocol for a Feasibility Randomized Controlled Trial
Source: JMIR Res Protoc. 2024 Apr 11;13:e57226. doi: 10.2196/57226 (PMC11046387; doi:10.2196/57226)
Supplement: Multimedia Appendix 2 [file resprot_v13i1e57226_app2.pdf]

uOBMRI Team Grant

Dear Dr. Ledoux,

We are pleased to inform you that your proposal for a uOBMRI Team grant has been funded at $75,000, through support from the uOBMRI at $50,000 and partner funding at $25,000. Your proposal was ranked at **3.98** (based on CIHR scoring) and was peer reviewed by experts in the field (please find reviewers' comments below).

Accepting these funds requires publication of project description and participation in community outreach events, including either engaging donors, public education or awareness events.

Six months after the end of the grant period (18 months), any unspent funds must be returned to the uOBMRI Research Fund, while any over-expenditure will be the responsibility of the researcher.

Congratulations on your success,
The uOBMRI Team

uOBMRI Team Grant Review

Reviewer #1

1. *The research team*: Expertise, is it multidisciplinary, is there an early career investigator, trainees, dose it engage patients, caregivers and/or community members.
    a. Large interdisciplinary team that satisfies all the requirements of the competition. A third party app developer (Mobio) is also providing software development expertise, critical to the project.
    b. As the study deals directly with patients (concussed youth), engagement with patients and caregivers is built into the project.

2. *Novelty and/or impact of the research*: Does this project have the potential to evolve into a future network or research program?
    a. The project extends and builds upon two separate CHAMO and ECR Growth Awards that the PI is overseeing. In these studies, the mobile app is applied and indices of QoL, emotional/cognitive symptoms are assessed. The proposed studies will use an MRI based approach to examine objectively indices of brain recovery post-concussion ± mindfulness-based intervention/training using the mobile app software. Objective verification of the clinical evaluation by MRI will ultimately propel the studies towards a larger CIHR-based project. Novelty is therefore somewhat limited in that the intervention is not entirely new, however the evaluation of the intervention is going to be more sophisticated and comprehensive.

3. *Research Approach and Feasibility*: Please comment on the strengths and weaknesses.
    a. Feasibilty is strong given the extensive expertise of the group. The assembled team is already studying groups of patients. The MBI interventions have already been successfully tested. The question remains as to whether this will be quantitatively detectable at the level of MRI – and whether other factors (e.g., severity of concussion, location of damage, age of individual 12yrs vs 18 yrs, sex differences) will be confounding for the limited cohort size.

4. *Knowledge mobilization plan*: Does it engage the community and is it feasible?
    a. Excellent dissemination plan that brings together a large number of community members and levels (including basic scientists/clinicians/technical staff/patients).

5. Other comments
    a. A project that builds upon an innovative intervention approach (MBI-based app) for recovery from concussions in adolescents. The team is excellent and ticks all the boxes for this competition. Novelty is held back slightly as the intervention exists, yet it will now receive objective evaluation by MRI.

Overall Score: 4.0

Descriptor	Range	Definition
Outstanding	4.5-4.9	The application excels in most or all relevant aspects. Shortcomings are minimal.
Excellent	4.0-4.4	The application excels in many relevant aspects, and reasonably addresses all others. Certain improvements are possible.
Good	3.5-3.9	The application excels in some relevant aspects, and reasonably addresses all others. Some improvements are necessary.
Fair	3.0-3.4	The application broadly addresses relevant aspects. Major revisions are required.
Poor	0.0-2.9	The application fails to provide convincing information and/or has serious inherent flaws or gaps.

Reviewer #2

1. ***The research team***: Expertise, is it multidisciplinary, is there an early career investigator, trainees, dose it engage patients, caregivers and/or community members.
   • The PI is an early career investigator at CHEO with research expertise in neuroimaging. She has an acceptable publication record at this stage of her career and has a good amount of funding. The co-investigators have expertise in neuroimaging, neuropsychology, psychiatry, emergency medicine, occupational therapy, and pediatric concussion, with one co-applicant holding a CRC I in pediatric concussion. The co-investigators include one early career researcher- and most are mid-career or senior researchers. One co-investigator has some experience with mindfulness research. There does not appear to be any expertise evaluating the benefits of digital therapies in general or for concussion in particular.
   Rating. 4.0

2. ***Novelty and/or impact of the research***: Does this project have the potential to evolve into a future network or research program?
   • The PI is requesting money to top up funds she has received for a pilot neuroimaging study on the effects of a newly developed mindfulness-based intervention (MBI) smartphone app for adolescents with concussion. Although the majority of people recover from a concussion, the app is intended to be a preventive intervention to reduce risk of developing PPCS. The app could potentially provide a low-cost approach to disseminate a brief mindfulness program that could improve outcomes in adolescents with mTBI. The applicants are mainly interested in determining if the 4-week MBI-app improves the default mode network of the brain. However, to be able to realistically evaluate neural mechanisms, evidence is first needed to show this app has clinical efficacy. This app has not been tested in rigorously designed RCTs; hence, there are no data available to suggest the app, which provides a very low dose of mindfulness training, will significantly improve psychological and neurocognitive outcomes in concussed adolescents. It is difficult (in my opinion) to justify a team grant of $100,000 to support a fairly large neuroimaging study of an app with no established efficacy, no compliance data, and no data on user experience. If the app proves to be ineffective, its effect on the brain, although interesting, would

have little impact on the clinical management of adolescents following a concussion. The team should first demonstrate that their MBI-app improves **e**motional and cognitive functioning before embarking on research exploring possible neural mechanisms. Even intensive mindfulness training with MBSR and MBCT has no demonstrable effect on attention and executive functioning and effects on psychological symptoms are generally small. Further, most neuroimaging findings of mindfulness have not been replicated. So, the idea that a small dose of mindfulness training can significantly impact the brain may be overly optimistic. The applicants have received pilot funding from CHEO to test the efficacy of this novel app in concussed adolescents, as well funds to assess the impact of the app on the brain. I would be more enthusiastic about this project if the applicants publish very promising pilot data of the app's efficacy. Rating: 3.4

3. *Research Approach and Feasibility*: Please comment on the strengths and weaknesses.
   • Sixty adolescents will be recruited from the ER at CHEO and randomized to either the mindfulness app plus usual care or usual care only. After 4 weeks, indices of functional connectivity during resting state fMRI will be measured. Additional secondary outcomes will also be obtained. Participants with a previous history of psychiatric hospitalization are excluded, but there is no mention if outpatients with concurrent psychiatric disorders who are treated with medication or other non-pharmacological interventions, or those with other neurological issues, are excluded. If they are included in the study, the sample will be too heterogeneous to make sense of the data and important differences may be blurred. At the ER visit, research assistants will inquire if participants engage in meditative practices like mindful yoga. So, this may not be a meditation naïve sample, which introduces another important confound.
   • My primary concern with this project is the lack of a credible control condition. While usual care might be a convenient control, it is not an adequate one to carefully test the benefit of their mindfulness app. Also, usual care can vary considerably from patient to patient. Currently, there is growing concern in the mindfulness research community about exaggerated claims of the benefits of mindfulness and the lack of high-quality studies. Experts in the field strongly advocate the use of a credible active control that is inert to mindfulness. Studies that have used a credible active controls general fail to find an effect of mindfulness training. The proposed research should use a credible app-based control to rule out the many non-specific factors that can contribute to outcome and to increase confidence in findings.
   • The applicants propose a 20% attrition rate. This seems low considering the high attrition rate with app/internet-based interventions in clinical samples, and for adolescents with mTBI (e.g. research on the SMART app for mTBI reported that 50% of enrolled adolescents did not complete the 4-week program). The applicants do not discuss how they will deal with compliance/engagement with the app. This is important, as studies of MBIs have found either some association between home practice and outcome (including neuroimaging results), or no association. Even people with minimal home practice have been found to improve- therefore positive effects are not necessarily attributable to the practice of mindfulness.
   • The rationale for the low practice dose was not well articulated; neuroimaging research on

mindfulness have been based on long term mediators or meditation
naïve participants who completed intensive training in mindfulness (i.e.. 8-week MBSR with considerable home practice and 1-day silent retreat). What evidence is there to support the hypothesis that a brief mindfulness intervention with a low practice dose can alter the brain, especially in individuals who have sustained a brain injury?
• MRI scans are usually obtained in mindfulness intervention research at baseline and post-intervention. In the proposed research, scans are obtained only at week 4, which corresponds with the time individuals are likely to be identified as having persistent symptoms. The lack of baseline scans precludes analysis of within-group changes and if between-group effects are found at week 4, it could simply be due to differences at baseline. Randomization does not assure equivalence of groups and looking at one time point is not especially informative.
• Finally, there is no adequate follow-up to determine if the effects of the app, if any, are sustained beyond the 4-week period.
Rating: 3.3

4. *Knowledge mobilization plan*: Does it engage the community and is it feasible? The KT plan is acceptable but is not particularly novel. Rating: 3.5

5. *Other comments*
Overall score=3.55

Reviewer #3

1. *The research team*: Expertise, is it multidisciplinary, is there an early career investigator, trainees, dose it engage patients, caregivers and/or community members. Yes. The PI is an early career investigator as are several other members of this multidisciplinary team that also includes an industry collaborator. A parent-engagement leader will assist with dissemination of findings to families.

2. *Novelty and/or impact of the research*: Does this project have the potential to evolve into a future network or research program? Yes. There is potential for this proposal to form the basis of a larger research program that likely would, in the first instance, be Ottawa based. There is no mention of multi-centre trials. The novel aspect of the work is the attempt to use quantitative neuroimaging outcomes as a means to assess the ability of different interventions, such as a mindfulness based training app, to improve recovery after concussion.

3. *Research Approach and Feasibility*: Please comment on the strengths and weaknesses. This is very strong team with considerable expertise in mindfulness training, brain imaging and concussion. They have partnered with a company to develop a mindfulness-based intervention (MBI) mobile app that can be used at home by adolescents that have experienced concussion to improve their recovery. The central hypothesis of the work is that a neural network, termed the default-mode network (DMN) is active at rest but deactivates when the brain has external demands. The problem in concussion is that the DMN is hyperactive and does not deactivate thereby interfering with cognition, attention and emotion. There is evidence that MBIs can

improve attentional focus, mood, and cognitive flexibility in concussed patients. There is limited data suggesting that DMN activity may be dampened by MBI. The proposed study will be the first to use both clinical and neuropsychological outcome measures (some of which are quite subjective) with powerful brain imaging methods (fMRI, arterial spin labeling and diffusion tensor imaging (DTI)) to gauge efficacy of the MBI. As such, the applicants will measure resting state functional connectivity (fMRI), changes in cerebral blood flow (CBF, arterial spin labeling) and white matter (WM) integrity (DTI). It is hypothesized that early MBI will reduce connectivity within the DMN. They will also explore whether the MBI also impacts CBF and WM structure. These imaging-based outcomes will be related to the more subjective, cognitive and psychological outcomes typically employed. Strengths: This is a novel, powerful approach addressing an important problem. The applicants have the necessary expertise to conduct these experiments. Minor Weaknesses: The parent-engagement leader, Kathy Leeder, is listed as an applicant but her name is not included in the "Expertise of the applicants" section on page 4. It is unclear what her background is and how she will spread information to families. The application contains a number of acronyms (CATT, OBI, ONF, Parachute) that are not defined requiring the reviewer to track them down.

4. *Knowledge mobilization plan*: Does it engage the community and is it feasible? Yes. They will publish results in the usual academic journals. Findings will be used to update current national and local care guidelines, including web-based platforms used by patients, caregivers, sports organizations.

5. *Other comments*

   Budget well justified

   Score: 4.4
